# Deep Ensemble Learning for Application Traffic Classification Using Differential Model Selection Technique

**DOI:** 10.3390/s25092853

**Published:** 2025-04-30

**Authors:** Ui-Jun Baek, Yoon-Seong Jang, Ju-Sung Kim, Yang-Seo Choi, Myung-Sup Kim

**Affiliations:** 1 Department of Computer and Information Science, Korea University, Sejong 30019, Republic of Korea; pb1069@korea.ac.kr (U.-J.B.); brave1094@korea.ac.kr (Y.-S.J.); jsung0514@korea.ac.kr (J.-S.K.); 2 Electronics and Telecommunications Research Institute, Daejeon 34129, Republic of Korea; yschoi92@etri.re.kr

**Keywords:** deep ensemble, model selection technique, application traffic classification, end-to-end ensemble learning, network management

## Abstract

As the Internet evolves, application traffic is becoming increasingly diverse and complex, leading network administrators to demand more accurate application traffic classification. Various deep learning-based application traffic classification methods have clearly achieved significant success, demonstrating superior classification performance compared to traditional heuristic classification approaches. However, achieving accuracy while maintaining time-efficiency and high generalization performance remains a challenge. We propose an end-to-end learning method that incorporates a model-selection-based ensemble mechanism to improve the performance–inference time trade-off of application traffic classifiers. Evaluated on two public datasets and one private dataset, our proposed method improves classification accuracy across all datasets while ensuring reasonable inference times compared to nine classification methods.

## 1. Introduction

Recent advancements in IT technologies, including cloud computing and AI applications, have led to the development of diverse applications and services, resulting in increasingly complex application traffic and a continuous rise in traffic volume. According to MindInventory, the enterprise software market is expected to exceed USD 401.6 billion by 2029, with a Compound Annual Growth Rate (CAGR) of 6.35%, while the global cloud computing market is projected to surpass USD 1266.4 billion by 2028, growing at a CAGR of 15.1% [1]. This trend is likely to accelerate further, considering the deep integration of AI applications and machine learning technologies in software development, as well as the growth of low-code and no-code platforms [2]. The acceleration in application and service development leads to an increase in network traffic volume. Gitnux predicts that global Internet traffic will grow at a CAGR of 24% from 2021 to 2026 [3], while Cloudflare states that global Internet traffic increased by 25% in 2023 [4]. Given the increase in diverse and complex applications and services, along with the growth in their traffic volume, research on accurate and fast application traffic classification has become essential.

Application traffic classification is the process of categorizing network traffic into various applications or services. It is one of the most crucial tasks in network management. Network administrators can utilize application classification results to perform the following tasks:

- **Traffic monitoring and optimization**: Network resources can be efficiently allocated by analyzing the traffic volume of specific applications.
- **Security enhancement**: Abnormal traffic or attack traffic can be detected to prevent or respond to network attacks.
- **Bandwidth management**: Bandwidth can be limited or prioritized for applications that use high bandwidth.
- **Network performance analysis**: The impact of specific applications on network performance can be analyzed and improved.
- **Regulation and audit**: Traffic records can be analyzed to meet legal or regulatory requirements.

Application traffic classification research has evolved from traditional methods such as port-based classification, deep packet inspection, and behavior-based analysis to techniques utilizing machine learning and deep learning. In particular, there have been numerous studies on application traffic classification using deep learning technologies such as CNNs [5,6], RNNs [7], and attention mechanisms [8]. Recently, with the advancement of LLMs, research on pre-training to capture prior knowledge of network traffic modality and fine-tuning to transfer learned knowledge to target domains [9,10,11] has been actively conducted, aiming for the following requirements and challenges [12]:

- **Effectiveness**: It should provide traffic visibility and accurately classify network traffic.
- **Deployability**: Traffic classification models should be deployable within network assets and constraints.
- **Trustworthiness**: The results of traffic classification should be reliable.
- **Robustness**: The model should continue to function properly despite changes in the network environment.
- **Adaptivity**: When adjusting classification tasks according to environmental changes, the classification model should be able to adapt to these changes.

While the advancement of deep learning technology has clearly achieved many successes in addressing the limitations of various traditional methods, including machine learning approaches, there are still limitations that need to be resolved. Overfitting and slow inference speeds are presented as representative limitations of deep learning-based approaches. The overfitting problem is a phenomenon where deep learning models are excessively optimized for training data, resulting in reduced generalization ability for new data. Overfitting can occur when the model is overly complex, or when noise features are included. In particular, unique identifiers unrelated to applications contained in network traffic packets (for example, the IP address field in the IP layer, the Server Name Indication field in the TLS layer, etc.) are highly likely to cause overfitting problems. Representative methods to solve the overfitting problem include early stopping, network reduction, expansion of the training data, regularization [13], and ensemble techniques.

**Figure 1:**
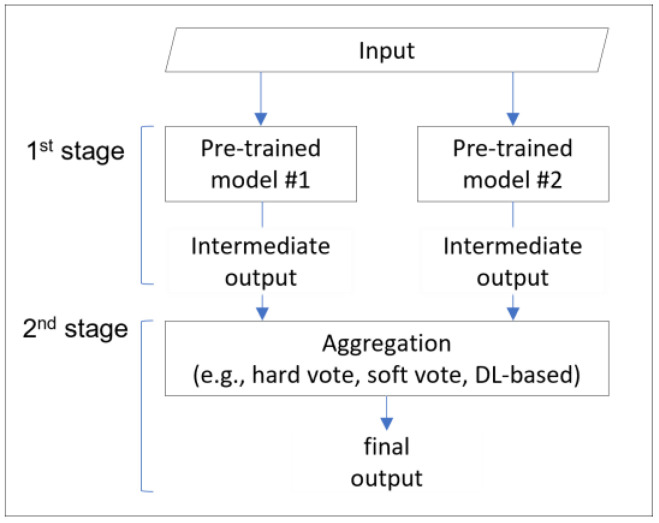
General process of ensemble techniques.

We chose the ensemble technique to address the overfitting problem and improve generalization performance. Ensemble techniques can effectively mitigate overfitting issues by combining multiple features or models to achieve higher performance, stability, and generalization ability than a single model. The general process of ensemble techniques is divided into two main stages, as shown in Figure 1. The first stage is the intermediate output generation process, where pre-trained intermediate classifiers process the inputs and generate intermediate outputs or temporary classification results. The second stage is aggregation and classification, where the output decision-maker (e.g., hard vote, soft vote) aggregates the intermediate outputs and generates the final output.

**Figure 2:**
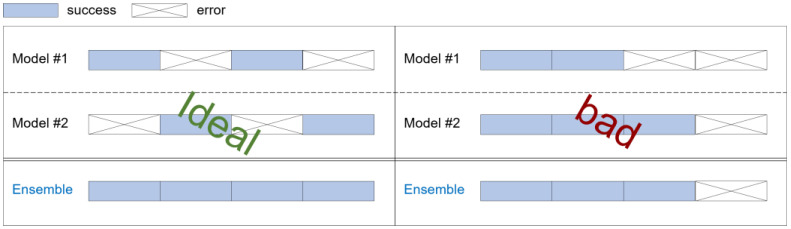
Ideal case and bad case of ensemble learning.

While there have been various studies attempting to improve generalization performance by utilizing ensemble techniques in multiple deep learning models, these also have limitations, one of which is slow inference time. In such ensemble approaches, all intermediate outputs must be generated before they can be aggregated and the final output produced, inevitably resulting in slow inference times. Even if the problem of slow inference time is solved by performing intermediate classifications in parallel, there is still the limitation of having to perform many computations. Another limitation is the diversity of learning, which refers to the differences between intermediate classifiers, and models are considered to be diverse when the errors for unseen instances differ between classifiers. Also, the success of ensemble learning heavily depends on the accuracy and diversity of the intermediate classifiers [14]. In other words, if the accuracy profiles of pre-trained models are similar, there may be data instances that can never be classified through existing output decision methods, as explained in Figure 2. When using an ensemble with two different models, in the ideal case (left), the errors of Model 1 and Model 2 can complement each other, resulting in high classification accuracy. However, in the bad case (right), the errors of Model 1 and Model 2 are not complementary.

We propose an end-to-end ensemble learning method based on a differentiable model selection strategy. The proposed method selects the intermediate classifier to generate the intermediate output based on the output logits of the model selector, and then sets that output as the final classification result. This model selection technique is similar to winner-takes-all [15] and has the advantage of being able to infer within a reasonable time, as it does not need to perform all intermediate classifications. Additionally, we adopt an end-to-end approach that learns the model selector and intermediate classifiers simultaneously rather than separately, and through two novel additional loss functions that control the model selection process, we induce competition among the intermediate classifiers and improve the learning diversity.

Following the introduction, Section 2 of this paper explains research on deep learning model ensembles and model selection techniques. Section 3 describes the proposed deep ensemble method, model structure, and loss functions. Section 4 demonstrates the effectiveness of the proposed method through datasets and various experimental results. Section 5 provides insights into the operating principle through a comparison with bagging, one of the existing ensemble techniques. Finally, Section 6 summarizes the conclusion, presents limitations, and suggests future research.

## 2. Related Works

### 2.1. Deep Learning-Based Application Traffic Classification

The history of deep learning-based application traffic classification is closely tied to the development of artificial intelligence and network traffic analysis. Early application traffic classification could easily categorize applications using well-known port numbers on the Internet, but the emergence of applications using dynamic ports revealed issues with inaccuracy and reliability. Subsequently, deep packet inspection (DPI), which examines packet contents, emerged but showed limitations due to traffic encryption and protocol encapsulation. To address these limitations, classification methods using statistical characteristics of traffic (packet size, inter-arrival time, etc.) were developed without considering the payload content. From the late 2000s, machine learning algorithms were introduced to traffic classification [16]. In machine learning-based classification methodologies, many studies have used supervised learning methods such as Support Vector Machine (SVM), K-Nearest Neighbors (KNN), and decision trees to learn and classify network traffic characteristics. These techniques primarily relied on feature engineering, so how the features were selected significantly impacted their performance. In the 2010s, the growth of deep learning technology brought significant changes to traffic classification. Deep learning provided the ability to automatically learn complex patterns from large-scale data, reducing dependence on feature engineering, and achieving success in various fields, including application traffic classification. Recently, research has been conducted to improve classification accuracy using large-scale pre-trained models [9,11,17] with strong generalization performance. While studies on model lightweighting are being conducted, considering the excessive size of these models, there is still a lack of research that improves the trade-off between performance and inference time itself. As we approach the commercialization of 6G networks beyond 5G, research is needed to accurately and quickly classify the complex and vast traffic of future Internet environments [18,19].

### 2.2. Application Traffic Classification Using Ensemble Techniques

This section introduces some studies that combine machine learning or deep learning models through ensemble techniques. Possebon et al. proposed a method to combine SVM, KNN, decision tree (DT), and Multi-Layer Perceptron (MLP) using voting, stacking, bagging, and boosting techniques [20]. Amin et al. proposed a method to independently train multiple CNN models and then combine the outputs of each model [21]. Ons et al. proposed a two-stage ensemble learning approach, using basic classifiers such as DT, Random Forest (RF), Adaboost, and XGBoost in the first stage, and then a DL-based meta-classifier in the second stage to combine the outputs of these basic classifiers for final classification [22]. Like these studies, the majority of research utilizing ensemble techniques in the field of application traffic classification still adheres to traditional aggregation strategies or deep learning-based output combination, mainly using machine learning models or simple deep learning models as intermediate classifiers. The reason these studies have to use lightweight classifiers is due to constraints on inference time or computational load, which arise because traditional ensembles require all intermediate classifiers to perform inference for aggregation and classification. In other words, in the existing ensemble structure where all intermediate classifications must be performed, there must be limitations in applying deep learning-based classifiers. Therefore, to fully combine deep learning technology and ensemble methods without constraints, research on efficient ensemble techniques is necessary.

### 2.3. Straight-Through Gumbel-Softmax

Integrating model selection into the deep learning process has traditionally been challenging due to the non-differentiability of categorical discrete variables. However, the emergence of the Gumbel-softmax [23] technique has made it possible to sample discrete variables within deep learning models. Gumbel-softmax is an extension of the Gumbel-max trick [24] through softmax, approximating the sampling of categorical variables in a continuous probabilistic manner, making it differentiable. The formula for Gumbel-softmax is as follows:

(1) $$y_{k}=\frac{exp((log\pi_{k}+g_{k})/\tau )}{\sum_{j=1}^{K}exp(log\pi_{j}+g_{j}/\tau )}$$

where $\pi_{k}$ represents the categorical distribution for the kth data instance, K is the number of classes, and τ is the temperature parameter. As τ decreases, the Gumbel-softmax distribution approaches a one-hot vector, and as τ increases, the distribution becomes more uniform and continuous; $g_{i}$ is the Gumbel noise sampled from a uniform distribution according to the following equation:

(2) $$g_{k}=-log\left(-\log\left(\mu_{k}\right)\right)$$

This study uses the straight-through Gumbel-softmax, which maintains differentiability while generating discrete samples. Straight-through Gumbel-softmax generates discrete samples using argmax in the forward pass and propagates the loss according to the Gumbel-softmax gradient in the backward pass, which can be expressed by the following equations:

(3) $$\frac{\partial L}{\partial \theta_{i}}=\sum_{k=1}^{K}\frac{\partial L}{\partial y_{k}}\frac{\partial y_{k}}{\partial \theta_{i}}$$

where L is the loss function and $\theta_{i}$ is the ith logit parameter.

### 2.4. Model Selection Mechanism

The model selection mechanism is a method for selecting one of the logits output by each model when there are multiple candidate models, and then choosing it as the final result of the ensemble.

**Figure 3:**
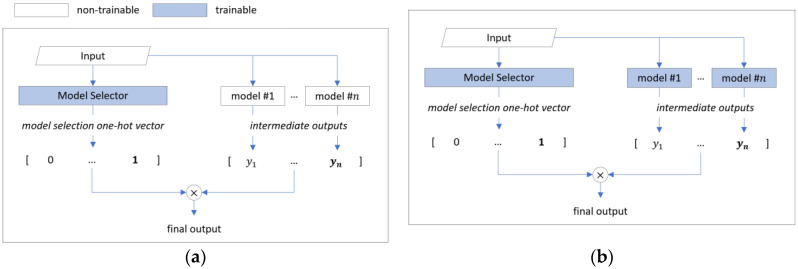
Ensemble deep learning model based on model selection technique: (**a**) Overview of existing ensemble based on model selection technique (Kotary, et al.). (**b**) Overview of end-to-end ensemble based on model selection technique (proposed).

This process is illustrated in Figure 3a. In the model selection mechanism, each candidate model can have diverse accuracy profiles, and they may complement each other across the test sample distribution. This approach was initially proposed to optimize channel selection [25] and feature selection processes [26], and it is being utilized in areas such as neural architecture search [27]. In particular, differentiable model selection has the potential to create superior models and efficient ensembles by automating the optimal model selection for classifying specific input samples.

Recently, Kotary et al. proposed E2E-CEL (end-to-end combinatorial ensemble learning) [28], which ensembles pre-trained classifier outputs using a differentiable model selection technique, as illustrated in Figure 3a. This approach consists of three main stages:

- **Intermediate output generation**: Intermediate classifiers (ensemble agent models) receive data samples and generate intermediate outputs (agent predictions).
- **Model selection**: The model selector (selection net) receives data samples and generates a one-hot vector determining which model’s intermediate output to use.
- **Aggregation and classification**: The final output is generated by multiplying the intermediate classifier’s intermediate output by the model selector’s one-hot vector.

E2E-CEL performs classification tasks by combining pre-trained models and achieves improved classification performance. However, it does not process data from input to classification output within a single model, meaning that it cannot be considered to be fully end-to-end from an ensemble learning perspective. Additionally, since E2E-CEL independently trains intermediate classifiers, there is no collaborative or competitive mechanism during the training process. Consequently, if intermediate classifiers are structurally similar or rely on similar features, they inevitably produce correlated outputs. This limits error diversity, prevents the outputs from complementing one another, and may lead to the bad case shown in Figure 2.

The proposed method represents a fully integrated ensemble approach that combines both stages of traditional ensemble learning: intermediate classifier training (Stage 1) and aggregation (Stage 2), as shown in Figure 3b. In this end-to-end ensemble learning, the weights of intermediate classifiers can be dynamically adjusted based on model selection outputs. Additionally, two novel loss functions—selection balance loss and selection freeze loss—regulate competition or collaboration between models. These mechanisms enhance error diversity in the ensemble model and optimize the classification outcomes.

## 3. Deep Ensemble Using the Model Selection Technique

### 3.1. Overview of the Deep Ensemble Process

**Figure 4:**
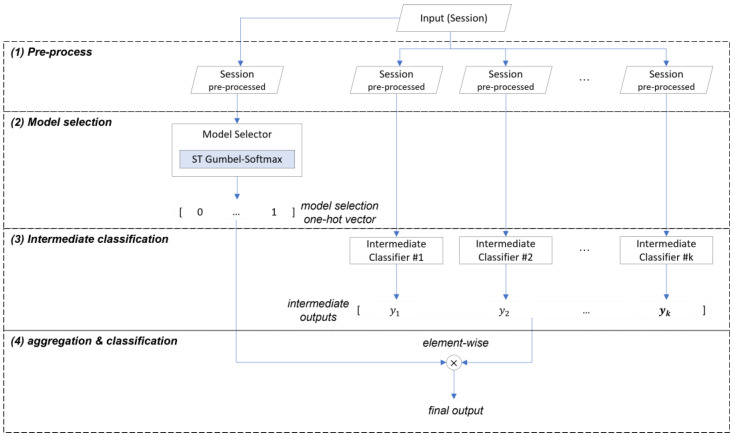
The four stages of the deep ensemble model.

This section provides an overview of the deep ensemble model structure and a summary of each learning stage. The deep ensemble model consists of four stages, as shown in Figure 4, and receives traffic session data as inputs, which it processes through each stage, and then outputs the probability values of belonging to each class as the final classification result.

The first stage is data preprocessing, which transforms the input to suit each intermediate classifier. In this study, some publicly available models from existing research were used as intermediate classifiers, and the input was transformed according to the input requirements of each classifier. In the proposed method, there are no duplicate models within the ensemble, forming a heterogeneous combination where diverse input forms and model structures can improve the classification performance by increasing the error diversity [22]. The second stage is the model selection process, where the model selector receives the preprocessed session, extracts features, and outputs the model selection result, which is the probability that each intermediate classifier’s output will be selected as the final output. The model selection result is a one-hot vector of size equal to the number of intermediate classifiers. The third stage is the intermediate classification process, where each intermediate classifier receives the preprocessed session, extracts features, and generates an intermediate output. The final stage is the aggregation and classification process, which multiplies the model selection result by the intermediate outputs and then sums them to generate the final output, which is a vector of probabilities that the session belongs to each class.

### 3.2. Baselines for Intermediate Classifiers

We selected five publicly available models for configuration and evaluation of the proposed deep ensemble method. The first model was the 2D-CNN [5], which was the first attempt to apply a representation learning approach to malicious traffic classification using raw traffic data. It proposed three models and showed superior performance compared to existing methodologies. The second model was the 1D-CNN [6], which was the first attempt to apply an end-to-end classification method in the field of encrypted traffic classification. It validated the method’s effectiveness using the public dataset and showed superior performance in 11 out of 12 evaluation metrics compared to existing methodologies. The third and fourth models were the HAST-IDS [7], which is divided into two sub-models. Both sub-models use a CNN to learn low-level spatial features of network traffic and LSTM networks to learn high-level temporal features. The difference between the first sub-model HAST-1 and the second sub-model HAST-2 is the presence or absence of the LSTM network. The HAST-IDS model was evaluated using two public datasets and showed excellent performance compared to existing methods. The fifth model was the SAM model [8], which uses a self-attention mechanism to classify application traffic. SAM demonstrated high classification performance while ensuring real-time operation by using smaller inputs compared to existing models, and it also provided interpretability.

In the proposed method, a corresponding intermediate classifier must be selected each time a data instance is classified, which can be a significant overhead in terms of inference time. To minimize this overhead, we adopted SAM as the basic structure of the model selector, which has fast inference time while maintaining excellent classification performance. The model selector is structured by adding the straight-through Gumbel-softmax after the SAM baseline. It receives the output of the fully connected layer located after SAM as an input, and it outputs a one-hot vector for model selection with a size equal to the number of intermediate classifiers.

### 3.3. Loss Functions for Improving Error Diversity and Learning Stability

This section proposes two additional loss functions besides cross-entropy for end-to-end learning to improve error diversity: selection balance loss, and selection freezing loss. The final loss is a weighted sum of these three losses, expressed by the following equation:

(4) $$L_{total}=L_{CE}\cdot W_{CE}+L_{SB}\cdot W_{SB}+L_{SF}\cdot W_{SF}$$

The first loss function is cross-entropy loss, which calculates the difference between the predicted class probabilities and actual class probabilities, aiming to improve the accuracy of the final output. The equation is as follows:

(5) $$L_{CrossEntropy}=-\sum_{i=1}^{N}\sum_{j=1}^{K}y_{ij}\cdot log(p_{ij})$$

where N is the number of samples, K is the number of classes, $y_{ij}$ indicates whether the ith sample belongs to the jth class, and $p_{ij}$ is the predicted probability that the ith sample belongs to the jth class. The cross-entropy loss is backpropagated to both the model selector and the intermediate classifiers.

The first additional loss function is the selection balance function, proposed to address the selection monopoly problem. The loss value transmitted to each intermediate classifier varies according to the model selector’s output, due to the application of the straight-through technique. In other words, the selected model receives a higher proportion of loss compared to unselected models, resulting in a higher learning rate. This characteristic causes a selection monopoly problem and hinders the normal ensemble of the model. The selection balance function aims to control the selection frequency of each model by the model selector, solving the selection monopoly problem and improving the error diversity. The data monopoly problem occurs when deep learning models with initially high learning rates monopolize data instance allocations, as they learn in the direction of minimizing loss according to weight changes during the training process. When training a single model, the data allocation results vary depending on each model’s learning speed. According to our experiments, the comparison of learning speeds between models is as follows:

(6) 2D-CNN≈1D-CNN≈SAM≫HAST-1>HAST-2

**Figure 5:**
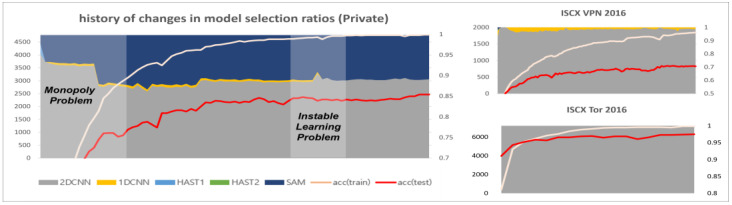
The monopoly problem and the unstable learning problem.

The 2D-CNN, 1D-CNN, and SAM have faster learning speeds compared to HAST. When training using only cross-entropy, without additional loss functions, CNN models and SAM, with their faster learning speeds, are allocated all of the data, as shown in Figure 5. Each plot in Figure 5 represents the history of changes in model selection ratios for each dataset, with the x-axis showing the training epochs and the y-axis showing the sum of selection counts for each model. At the beginning of training, CNN models, HAST-1, and SAM are observed to be allocated data and trained, but HAST-1, with its slower learning speed, is gradually not allocated data, and towards the end of training, the CNN models and SAM are observed to be allocated all of the data. This occurs similarly in other datasets.

The selection balance loss is the standard deviation of the sum of selection counts for each model, expressed by the following equation:

(7) $$L_{SB}=\sqrt{\frac{1}{M}\sum_{j=1}^{M}{\left(c_{j}-\mu \right)}^{2}}\;c=\sum_{i=1}^{n}y_{i},\;\;\;\;\mu =\frac{1}{M}\sum_{j=1}^{M}c_{j}$$

where $y=\{y_{1},\;y_{2},\;\ldots ,\;y_{n}\}$ is the set of one-hot vectors for model selection across n data instances, M is the number of models, $y_{i}=R^{M}$ is an M-dimensional one-hot vector for the ith data instance, $c_{j}$ is the sum of selection counts for the jth model, μ is the average of selection counts for each model, and the $L_{SB}$ is the standard deviation of the sum of selection counts for each model. The core idea of the selection balance loss is to enable diverse learning by guaranteeing a minimum learning opportunity for each intermediate classifier, and the experimental results confirmed that it functions as intended in its design.

The second additional loss function is the selection freezing function, proposed to address the learning instability problem. The learning instability problem occurs when the model selection results change drastically due to changes in the weights of the model selector during the learning process. An example of the learning instability problem is shown in Figure 5. The model selector is a function that finds the optimal solution for the data allocation problem for each classifier, and it is recommended to vary the data allocation information diversely in the early stages of learning. However, if the allocation information changes significantly when each intermediate classifier is optimized for the allocated data, it can cause great confusion to the distribution that the classifier has already learned in the latter part of the training. The selection freezing loss is calculated as the inverse of the deviation of the sum of model selection history per data instance, and the calculation formula is as follows:

(8) $$L_{SF}={\left(\frac{1}{N}\sum_{i=1}^{N}\sigma_{i}\right)}^{-1}\;S_{i,j}=\sum_{e=1}^{E}S_{i,j,e}\;\sigma_{i}=\sqrt{\frac{1}{M}\sum_{j=1}^{M}{\left(S_{i,j}-\mu_{i,j}\right)}^{2}}$$

where M is the number of models, N is the number of data instances, E is the number of epochs accumulated in the memory, $S_{i,j,e}\in R^{M}$ is the one-hot vector of model selection for the ith data instance in the eth epoch, $S_{i,j}$ is the number of times the ith data instance was selected in the jth model, $\sigma_{i}$ is the standard deviation of the number of model selections for the ith data instance, and $L_{SF}$ is the inverse of the average of the standard deviations of selection history for each data instance. The core idea of the selection freezing loss is to give intermediate classifiers sufficient time to fine-tune on the allocated data in the latter part of the training, and the experimental results confirmed that it functions as intended in its design.

## 4. Experiments and Evaluation

### 4.1. Datasets

This section provides a description of the datasets used to validate the proposed method. Three datasets were used: one public dataset and two private datasets. An overview of the datasets is presented in Table 1. Each dataset was split into training and test datasets at an 8:2 ratio and used for training and testing after undergoing refinement and preprocessing. Refinement is the process of removing sessions that may interfere with model training, eliminating sessions that meet the following conditions:

- Incomplete session: Elimination of TCP or TLS sessions without a hand-shake process
- Non-payload session: Elimination of TCP sessions that do not contain a payload
- Unrelated protocol sessions: Elimination of sessions from protocols considered to be unrelated to applications, such as DNS, LLMNR, MDNS, etc. [31].

**Figure 6:**
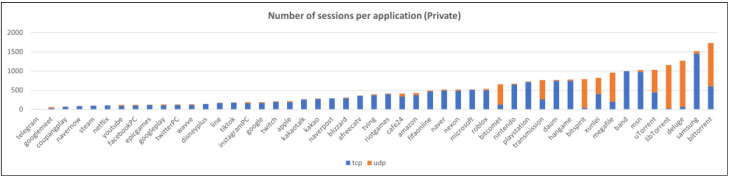
The number of sessions per application (private dataset).

**Figure 7:**
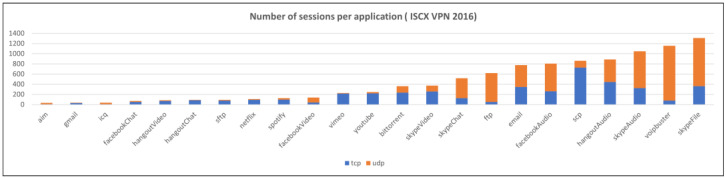
The number of sessions per application (ISCX VPN 2016).

**Figure 8:**
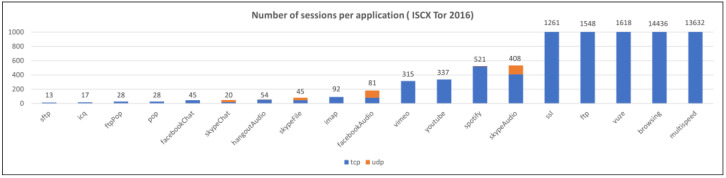
The number of sessions per application (ISCX Tor 2016).

Preprocessing is the process of removing IP headers and TCP/UDP headers that may cause biased learning or overfitting when converting each session into model inputs. The distribution of the number of sessions for each dataset and application after refinement and preprocessing is shown in Figure 6, Figure 7 and Figure 8.

### 4.2. Overall Comparisons with Other Methods

Overall, the proposed methodology leads to improved accuracy while ensuring reasonable inference speed compared to the other methods. In the private dataset, there was a 1.8% p improvement in classification accuracy compared to HAST-1, which showed the highest performance among baselines, and it demonstrated 1.8 times faster inference speed in terms of inference time. Compared to XGB, which showed the best performance among the comparison models, the accuracy improved by 0.5% p, but XGB showed overwhelming performance in terms of inference speed. However, in the ISCX Tor dataset, XGB showed low performance, while the proposed methodology showed the best performance. The traditional hard vote ensemble showed low performance except for ISCX Tor 2016, and the ensemble of pre-trained models (Kotary, et al.) did not show superior performance on all datasets. The proposed method showed the highest performance on all three evaluated datasets and guaranteed a reasonable inference time compared to other methodologies (Table 2).

### 4.3. Analysis of Training History Based on the Application of Loss Functions

**Figure 9:**
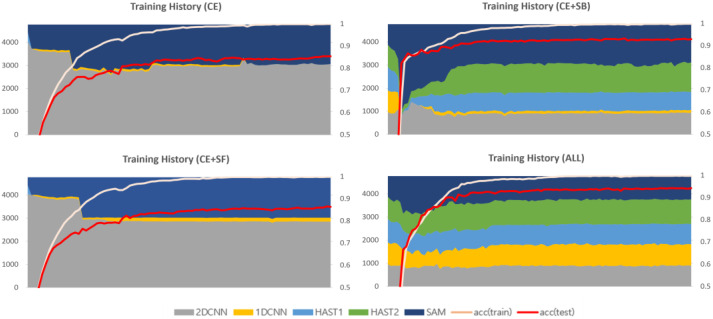
Training history based on the application of loss functions (private).

**Figure 10:**
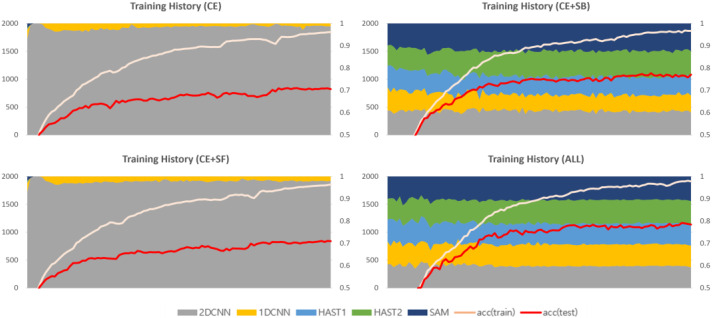
Training history based on the application of loss functions (ISCX VPN 2016).

**Figure 11:**
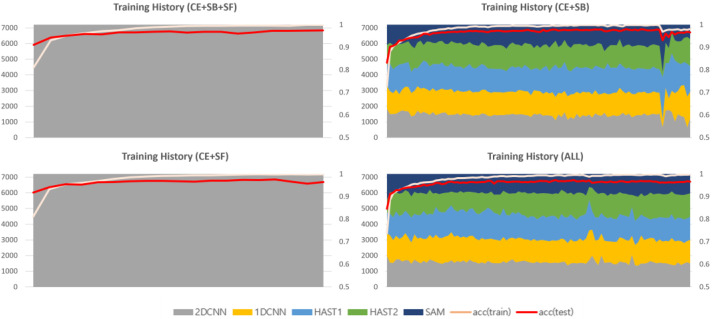
Training history based on the application of loss functions (ISCX Tor 2016).

This section provides a comparative analysis based on loss function weights, with comparison results for each dataset shown in Figure 9, Figure 10 and Figure 11. In the results for the private dataset, when only cross-entropy loss was used, only the initial HAST-1, SAM, and 2D-CNN participated in inference. When selection balance was added, the initial intermediate classifiers were allocated data evenly, and this result persisted until the latter part of training. Finally, when selection freeze was also applied, we can see that the model selection results did not change significantly in the latter part of training, indicating stable learning. Similar differences can be observed for the other datasets. In conclusion, the two proposed additional loss functions operate appropriately according to their design intentions. SB improves learning diversity by encouraging collaboration among all intermediate classifiers, while SF enhances learning stability by limiting model selection changes in the latter part of training.

### 4.4. Comparative Analysis of Loss Function Weights

**Figure 12:**
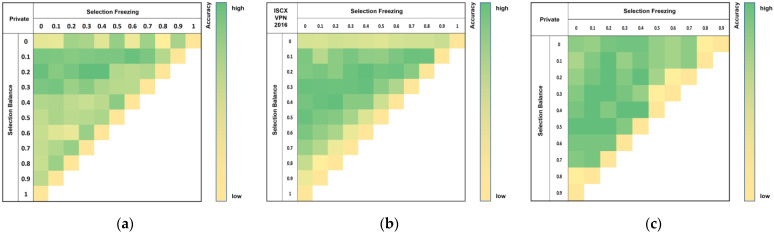
Comparison of accuracy based on loss function weights: (**a**) private dataset; (**b**) ISCX VPN 2016; (**c**) ISCX Tor 2016.

This section provides a comparative analysis based on loss function weights, and the comparison results are shown in Figure 12. After analyzing the results from all datasets comprehensively, it is appropriate to set the selection balance loss to around 0.2 and the selection freeze loss to about 0.2–0.4. Although there are differences between datasets, cases where both the selection balance loss and selection freeze loss are applied within the appropriate range demonstrate high performance.

### 4.5. Analysis of Error Diversity

**Figure 13:**
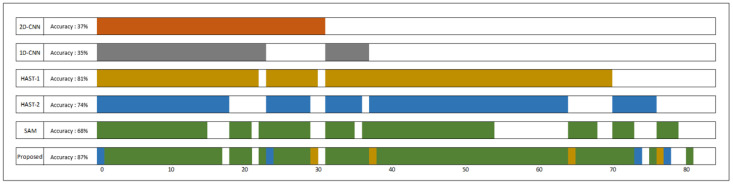
Comparison of coverage by model.

This section provides analysis results on error diversity, which is one of the conditions for successful ensemble learning. Table 3 shows the classification results according to the learning method of the base models. *Pre-trained* refers to the inference results on the entire test dataset after training each model separately, while “Proposed” shows the inference results on the entire test dataset after training using the proposed method. Compared to *Pre-trained*, the proposed method shows increased learning accuracy for *2D-CNN* and *1D-CNN*, but decreased accuracy for the remaining models. *Proposed (only assigned dataset)* represents the inference results only for the datasets assigned to each model from the test dataset. *2D-CNN*’s accuracy increased from 61.6% to 93.94%, and *1D-CNN* showed 100% accuracy for its assigned dataset, while the accuracy of *HAST-1*, *SAM*, and *HAST-2* decreased. The proposed method increases the error diversity of each model by appropriately dividing and allocating the test dataset to each model during the end-to-end learning process. This can also be observed in Figure 13, which compares the changes in model acceptance capacity.

Figure 13 shows the comparison results of model coverage for the first class (NaverNow) of the private dataset. The top 5 models are the baselines used as intermediate classifiers, and the results of the proposed method are located in the last row. Although *HAST-1* showed the highest accuracy among the base models, the proposed method assigned most of the dataset to *SAM*. Additionally, the proposed method was able to classify data that none of the existing models could classify. The proposed method enhances error diversity by dividing and allocating the test dataset appropriately to each model during the end-to-end learning process, which can also be observed in the comparison of changes in model coverage shown in Figure 13.

### 4.6. Comparison of Homogeneous and Heterogeneous Model Ensembles

This section provides an analysis of whether accuracy improvements were achieved when using only homogeneous models instead of five heterogeneous models. While the proposed method combines five heterogeneous models, this section presents experiments using ensembles of only homogeneous models for three lightweight model types (2D-CNN, 1D-CNN, SAM), as shown in Table 4. For 2D-CNN and 1D-CNN, accuracy improves as the number of models increases when combining homogeneous models. However, for SAM, the accuracy decreases. These results confirm that the proposed method follows the basic principle of ensemble learning, which improves performance by combining weak classifiers with suboptimal performance. On the other hand, the combination of three heterogeneous models showed higher accuracy compared to the combination of homogeneous models, with the best results achieved when using all five base models. This indicates that combining models with diverse input forms and structures helps improve generalization ability.

## 5. Discussion

**Figure 14:**
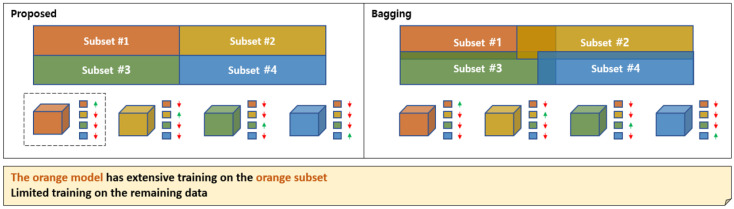
The differences between the proposed method and bagging, from the perspective of subset partitioning.

The proposed deep learning-based ensemble learning method has a similar operating principle to bagging and, thus, can be considered to have similar improvement effects. Bagging is one of the ensemble techniques that creates multiple random subsets from the training data and trains them, aiming to make the predictions more stable and consistent. A brief comparison is shown in Figure 14. In the bagging example in Figure 14, the entire dataset is divided into four subsets. The models corresponding to the subsets learn their assigned subsets, and finally, the outputs of all models are aggregated through voting or averaging. At this time, the subsets are not completely distinct, with some overlapping data instances between subsets, which means that each model learns some information from subsets that other models are learning, in addition to its assigned subset. This is indicated by arrows on the right side of each model, with up arrows meaning high learning rates and down arrows meaning low learning rates. The proposed method is similar to bagging in that it divides the entire dataset into as many subsets as there are models. Also, learning some information from subsets assigned to other models is similar, as it transmits continuous loss values rather than discrete ones based on the output of the model selector during backpropagation.

However, there are several differences from bagging, as shown in Table 5. First, while overlap between subsets is explicitly allowed in bagging, in the proposed method, the subsets are completely distinct during forward propagation, but they learn information from other subsets during backpropagation. In terms of subset creation, bagging randomly divides the subsets, while in the proposed method, the model selector performs this role, which is determined by learning. For aggregation, bagging uses voting or averaging, while in the proposed method, the model selector performs this role, which is similar to the “winner takes all” strategy. Other differences include that the proposed method does not perform out-of-bag validation, and the number of subsets and the number of divisions per subset are flexible, as they are determined by learning. In terms of feature selection, the proposed method does not automatically include features or perform validation processes. Regarding inference time, bagging requires outputs from all models, resulting in longer inference times, while the proposed method has balanced inference times due to the characteristics of the “winner takes all” strategy.

In conclusion, the proposed method can be seen to improve generalization ability through the process of subset division and learning, which is very similar to the mechanism by which bagging improves generalization ability. When examined in detail, there are many differences from bagging, and since bagging’s mechanism has clear advantages in improving generalization ability, it is thought that if research is conducted to additionally apply this to the proposed method, there is a possibility of further improving performance.

## 6. Conclusions

This study proposes a complete end-to-end deep ensemble method utilizing differentiable model selection techniques. To the best of our knowledge, this is the first attempt at a deep ensemble with model selection techniques applied. We have addressed the error diversity issue and inference time problem inherent in ensembles using pre-trained models through model selection techniques and end-to-end learning methods. The two additional functions that we have proposed operate appropriately, as intended. The proposed method enables faster and more accurate classification compared to existing approaches, achieving an average improvement of 4.8%p in accuracy and a 7× enhancement in inference time compared to transformer-based pre-trained models, a 4.9%p improvement in accuracy and a 2.6× enhancement in inference time compared to traditional ensemble methods, and a 2.3%p increase in accuracy and a 1.15× improvement in inference time compared to existing model-selection-based ensembles. On the other hand, while the proposed method is approximately 60 times slower in inference time on average compared to the XGB model, it has clear advantages. These include being sufficiently capable of real-time processing, demonstrating significant accuracy improvements on the ISCX Tor dataset, and offering flexibility for extension through different backbone networks, parameters, and learning strategies—unlike XGB. Although the proposed method has only been validated on three application traffic classification datasets, it is a general method that is not limited to specific fields and can be extended to other domains. There are three directions for future research:

1. Combining with the bagging mechanism, which is one of the existing ensemble methods, as mentioned in the Discussion.
2. Improving the combination method to address cases where the proposed method, despite its superior performance compared to existing methods, fails to classify some data instances that were classified by existing models.
3. Gaining a deeper understanding of the operating principles of the model selector and extending it towards Explainable Artificial Intelligence (XAI).

## Figures and Tables

**Table 1:** Overview of datasets.

Dataset	Publicly	#Task	#Applications	#Sessions	
				Raw	Preprocessed
Private	N	2	50	71,841	23,846
ISCX VPN 2016 [29]	Y	3	23	187,336	10,011
ISCX Tor 2016 [30]	Y	3	21	57,605	36,947

**Table 2:** Overall comparison with other methods.

Model	Private			ISCX VPN 2016			ISCX Tor 2016		
	Accuracy	F1-Score	Inference Time *	Accuracy	F1-Score	Inference Time *	Accuracy	F1-Score	Inference Time *
2D-CNN	61.6	60.8	113	64.9	64.6	47	96.5	96.3	175
1D-CNN	59.5	58.3	113	66.1	65.8	47	96.6	96.3	175
HAST-1	92.5	92.5	1646	73.1	73.1	691	97.0	96.8	2550
HAST-2	91.9	91.9	2384	69.9	70.1	1000	96.4	96.1	3693
SAM	92.0	92.0	146	71.9	71.9	61	94.2	93.8	226
XGB	93.6	93.6	**15**	79.1	73.1	**6**	90.0	87.1	**23**
ET-BERT	91.8	91.3	6663	70.9	70.9	2794	94.4	94.2	10,321
Hard vote	87.0	87.1	2384	72.0	73.1	1000	97.7	97.5	3693
Kotary et al.	93.1	93.1	1015	74.1	74.1	486	97.4	97.4	1423
Proposed	**94.3**	**94.0**	908	**79.2**	**79.1**	371	**98.0**	**97.9**	1374

* Inference time represents the total time (in milliseconds) taken to classify all data instances.

**Table 3:** Classification results according to the learning method of the baselines.

	2D-CNN	1D-CNN	HAST-1	HAST-2	SAM
Pre-trained	61.6	59.5	92.5	91.9	92
Proposed (Entire test dataset)	67.1	65.9	91.5	91.5	91.3
Proposed (Only assigned dataset)	93.94	100	99.3	77.3	93.2

**Table 4:** Comparison of homogeneous and heterogeneous model ensembles.

#Models	Accuracy				
	Homogeneous			Heterogeneous	
	2D-CNN	1D-CNN	SAM	2D-CNN 1D-CNN SAM	ALL
1	61.6	59.5	92.0	92.8	**94.3**
2	88.1	83.4	91.4		
5	87.8	88.4	91.4		
10	90.6	88.0	91.7		

**Table 5:** Comparison of the characteristics of bagging with the proposed method.

Items	Proposed	Bagging
Overlap between subsets	Implicit	Explicit
Subset creation	Model selector	Random
Aggregation strategy	Winner takes all Model selector	Vote, averaging
Out-of-bag strategy	Absent	Present
Number of subsets	Variable	Fixed
Number of divisions per subset	Variable	Fixed
Number of features	Fixed	Variable
Inference time	Balanced	Inefficient

## Data Availability

The source of the private dataset is security and cannot be disclosed. ICSX VPN 2016 is available for download from https://www.unb.ca/cic/datasets/vpn.html (accessed on 16 February 2025), and ICSX Tor 2016 from https://www.unb.ca/cic/datasets/tor.html (accessed on 16 February 2025).
